# Utility of AD8 for Cognitive Impairment in a Chinese Physical Examination Population: A Preliminary Study

**DOI:** 10.1155/2014/804871

**Published:** 2014-11-10

**Authors:** Yue Xie, Ya Gao, Jianjun Jia, Xiaohong Wang, Zhenfu Wang, Hengge Xie

**Affiliations:** Department of Geriatric Neurology, Chinese PLA General Hospital, 28 Fuxing Road, Beijing 100853, China

## Abstract

*Objective*. To investigate the utility of AD8 for cognitive impairment in a Chinese physical examination population. *Methods*. Military cadres who took routine physical examination in Chinese PLA General Hospital from Jan 1, 2013, to Dec 31, 2013, were subjected to AD8 scale. Individual information such as age, gender, and education was also collected. All data were analyzed by SPSS 19.0. *Results*. 1544 subjects were enrolled in this study with mean age 75.4 ± 10.6 years. The subjects who scored 0 to 8 of AD8 scale were 1015, 269, 120, 60, 30, 14, 19, 8, and 9, respectively. Corresponding proportions were 65.7%, 17.4%, 7.8%, 3.9%, 2.0%, 0.9%, 1.2%, 0.5%, and 0.6%, respectively. The endorsement prevalence of 8 questions was 5.6%, 9.2%, 6.6%, 9.2%, 4.8%, 4.5%, 8.9%, and 24.1%, respectively. The endorsement prevalence of question 8 was significantly higher than others (*P* < 0.05). 260 subjects were scored equal to or greater than 2. The abnormal rate was 16.9%. All the participants were stratified into 9 groups by age; the prevalence of dementia was highly correlated with age (*P* < 0.01). *Conclusion*. AD8 scale is a convenient and effective tool for cognitive screening in routine physical examination population.

## 1. Introduction

The prevalence of dementia is increasing steadily in recent years in China with the increasing life expectancy. Dementia causes disability in older adults and is considered a global public health priority by the World Health Organization [1]. To address this serious public health concern, general population with mild clinical symptoms demands the need for early intervention and treatment [2]. However, one of the reasons why dementia remains underdiagnosed due to the lack of a brief and sensitive clinical instrument for the detection of cognitive impairment in primary healthcare settings.

Although there have been several epidemiological surveys about the prevalence of dementia in China for the past few years, these studies did not sufficiently detect dementia at the very mild stage in primary care [3–5]. The reason is that cognitive screening instruments widely used in these studies such as the mini-mental state examination (MMSE) [6] and Montreal cognitive assessment (MoCA) [7] have limitations. MMSE has been reportedly poor in detecting mild cognitive impairment (MCI) because of ceiling effects, particularly in highly educated individuals [8]. Additionally, data showed that MMSE would fail to detect 85% of the individuals who were clinically rated as demented [9], while MoCA [7] requires trained staff and takes over more time (about 10 to 15 minutes) to administer, which is inappropriate for use in primary care settings. Given the limited duration of visits available to physicians in primary care centers, a brief screening tool capable of screening dementia, especially in its early stages, is necessary to meet wider needs of the community.

AD8 is a brief informant-based assessment developed by Washington University in St. Louis, which is shown to be sensitive to early cognitive change in a general population, even at its very mild stage [10, 11]. The AD8 is comprised of 8 items, follows a yes (1) or no (0) format, and takes approximately 3 minutes to complete. AD8 has been translated into Chinese and validated in Taiwan [12, 13] but is not used extensively in mainland of China.

In this study, we preliminary discussed the application of AD8 in healthy check-up sample of Chinese PLA General Hospital to detect cognitive disorders at the earliest possible stage.

## 2. Method

### 2.1. Study Participant

Military cadres who took routine physical examination in check-up center of Chinese PLA General Hospital from Jan 1, 2013, to Dec 31, 2013, were subjected to AD8 scale. 1544 of 2109 subjects volunteered to take a survey of AD8 in this study with mean age being (75.4 ± 10.6) years old. The AD8 was completed by the knowledgeable informant (usually a spouse or close family member) or participant in the absence of an informant.

### 2.2. The Training of Interviewers

All of our interviewers were experienced clinicians or senior nurses. Before their administration of AD8 to the people in the physical examinations, they had to receive a series of training courses with regard to dementia-related topics and to practice the administration of AD8 with the general population by working with experienced interviewers and physicians with internships.

### 2.3. Administration of the AD8

The AD8 was administered in Chinese version. The AD8 contains 8 questions that ask the informant to rate change in memory, problem-solving abilities, orientation, and daily activities and respondents indicate their endorsement as yes (1) or no (0) [11, 14]. The total AD8 score is generated by summing the number of the items responded to with “yes” and score ≥ 2 suggests cognitive impairment [9–11, 14]. The interviewers conducted interviews face-to-face with the participant and an informant in a room apart from the patient and the time to complete the AD8 and collected individual information such as age, gender, and education. The Chinese PLA General Hospital approved all procedures.

### 2.4. Retrospective Clinical Analysis

The clinical materials of AD8 ≥ 2 cases in our study were analyzed retrospectively. Diagnoses ranging from no dementia to all forms of dementia such as MCI, AD, vascular dementia (VaD), and Parkinson's disease dementia (PDD) were collected.

### 2.5. Statistics

All analyses were conducted using SPSS (version 19.0 for Windows, SPSS Inc., Chicago, IL, USA). Descriptive statistics were used to characterize. The groups were compared using *t*-tests for quantitative variables and chi square tests of independence for categorical variables. Spearman correlations were used to examine strengths of association. All tests were leveled for a 0.05 significance.

## 3. Result

In total, 1544 of 2109 participants volunteered to take a survey of AD8, with participation rate 73.2% and mean age 75.4 ± 10.6 years old (47–99 years old); the questionnaire was well accepted and readily answered. The characteristics of the samples are presented in Table 1. Most of participants were male predominant, nearly 97%. Three categories were used to reflect level of education: 56% of participants had low education (<9 years), 8% had moderate education (10–12 years), and 36% had normative education (>12 year).

The subjects who scored 0 to 8 of AD8 scale were 1015, 269, 120, 60, 30, 14, 19, 8, and 9, respectively. Corresponding proportions were 65.7%, 17.4%, 7.8%, 3.9%, 2.0%, 0.9%, 1.2%, 0.5%, and 0.6%, respectively. 260 subjects were scored equal to or greater than 2, and the ratio of suspected dementia patients was 16.9% (Table 2) with their mean AD8 score being 3.3 ± 1.7, mean age being 82.8 ± 8.5 years old (Table 3). Besides, retrospective analysis of cases in suspected demented group showed that 25% (65/260) of cases were AD/MCI, 5.4% (14/260) were PDD/VD, and 69.6% (181/260) were undiagnosed dementia (Table 3).

The prevalence of endorsement of each question in AD8 in all participants was 5.6%, 9.2%, 6.6%, 9.2%, 4.8%, 4.5%, 8.9%, and 24.1%, respectively. The endorsement rate of question 8 was significantly higher than others (*P* < 0.05). Besides, the endorsement rate of each question in AD8 and AD8 total score was significantly different between suspected demented and nondemented participants (*P* < 0.001 for each comparison of AD8 question and AD8 total score). In demented group, AD8-8: consistent problems with thinking and/or memory were most frequently reported with its prevalence: 83.1%, and then followed the AD8-7: difficulty remembering appointment with its prevalence: 47.3%. In nondemented group, AD8-8 also has the highest endorsement rate 12.1%, followed by AD8-4: trouble learning how to use a tool, appliance, or gadget with its prevalence: 2.6% (Table 4).

The mean age between suspected demented (82.8 ± 8.5) and nondemented subjects (73.9 ± 10.4) was significantly different (*P* < 0.001). All the participants were stratified into 9 groups by age; the prevalence of dementia was highly correlated with age (*P* < 0.001, Table 5).

## 4. Discussion

Using brief informant interviews enabled us to detect the subjects at risk to develop dementia and its earliest stages who may be further more amenable to intervention [14]. Particularly in the healthy check-up group, AD8 may gather relevant data as an aid for further examination due to the quick and safe questionnaire. The AD8 is highly correlated with gold standard evaluations including the CDR and neuropsychological testing and with imaging and cerebrospinal fluid biomarkers of AD [14, 15].

In our study, 1544 of 2109 participants volunteered to take a survey of AD8, with rate of response 73.2%, suggesting AD8 was well accepted by the participants. The participants administrated the AD8 were not entirely informants, part of them were patients themselves in all interviews. However, the AD8 can be used as a patient interview in the absence of an informant [10] and can be administered in person or by phone [14].

Our samples were male predominant (97%) due to the special characteristics of gender in army, and this does not represent the gender ratio in China. However, given that the questions of AD8 do not cover activities or functions which are specific to any gender, and gender did not seem to have differences in the validation studies of the AD8, it is unlikely that gender may influence the responses to the AD8 [14]. Our study has included more than half of the sample with less than 9 years of education. However, AD8 is less apt to be biased by education, or ethnicity; it may be considered an advantage to recognize dementia in lower educated samples [11, 16, 17]. The mean AD8 score of these suspected demented subjects (AD8 ≥ 2) in our study, 3.3 ± 1.7, was higher than the score, 2.9 ± 1.3, previously reported in Taiwan [13]. A reasonable explanation for this difference may be related to the sample characteristics of age; the mean age of 75.4 ± 10.6 in our study was older than the age of 66.9 ± 10.2 in Taiwan [13].

Our data show that the dementia prevalence of 16.9% was higher than the prevalence of 5.14% reported in China [5]. Part of the reason which can be explained is that the sample of our study was not population based; selection biases may influence the accuracy of results. However, this study did not focus on the diagnosing of dementia, but the screening, which helps us discriminate nondemented individuals (CDR 0) from those with even mild forms of cognitive impairment (CDR 0.5 or greater). Besides, retrospective analysis of cases in suspected demented group (AD8 ≥ 2) showed that 69.6% of suspected demented individuals had not received a dementia diagnosis. It is possible that with longitudinal follow-up, these individuals undiagnosed dementia will go on to develop cognitive impairment.

Consistent with previous study, in the prevalence of each subitem in AD8 subitems, we have found that AD8-8 (consistent problems with thinking and/or memory) and AD8-7 (difficulty remembering appointment) were the top two commonly endorsed items in the demented group, while reported in <13% of control individuals [13]. Such findings also underlined the importance of memory impairment in the detection of very mild dementia, especially in AD. In the nondemented group, AD8-4, trouble learning how to use a tool, appliance, or gadget, was the second most commonly endorsed. This question was not perfectly understood by the participants, because some of them reported that certain items, such as using a computer, were not really part of the daily activities.

We found that the prevalence of dementia had a significant positive correlation with age. Older age increases the likelihood of obtaining higher dementia prevalence. Age is the best-studied and strongest risk factor for dementia. The age effect observed in this study could be explained by the increasing rate of dementia in the older age groups.

There are several limitations in the present study. First, the sample of our study was not population based; selection biases limit generalization of the results. Hence, our study cannot reflect the real prevalence of current dementia in mainland of China. Second, in spite of the fact that 30.4% (79/260) suspected demented participants received the clinical diagnosis of dementia by retrospective analysis, the remaining undiagnosed dementia individuals in suspected demented group were not performed longitudinal follow-up to confirm the validity of AD8.

To the best of our knowledge, this is the first screening study to detect very mild dementia with AD8 in mainland of China. AD8 might be more brief, quick, and acceptable screening instrument for early dementia in the healthy check-up group. A further study with longitudinal follow-up to confirm the validity of AD8 is encouraged.

## Figures and Tables

**Table 1:** The basic information of participants.

	*n*	%
Total	**1544**	**100%**
Sex		
Male	1498	97%
Female	46	3%
Age (y)		
<50	12	0.8%
50–59	84	5.4%
60–64	198	12.8%
65–69	185	12.0%
70–74	264	17.1%
75–79	179	11.6%
80–84	217	14.1%
85–89	286	18.5%
≥90	119	7.7%
Education level (y)		
<9	865	56%
10–12	124	8%
>12	555	36%

**Table 2:** Characteristic of AD8 score.

Score	*n*	%
AD8 < 2	1284	83.1%
0	1015	65.7%
1	269	17.4%
AD8 ≥ 2	260	16.9%
2	120	7.8%
3	60	3.9%
4	30	2.0%
5	14	0.9%
6	19	1.2%
7	8	0.5%
8	9	0.6%
Total	**1544**	**100%**

**Table 3:** Characteristic of participants suspected dementia^*^.

	Total (*n* = 260)
Mean AD8 score	3.3 ± 1.7
Mean age (y)	82.8 ± 8.5
Disease constitution	
AD/MCI	65, 25%
PDD/VaD	14, 5.4%
Undiagnosed dementia	181, 69.6%

^*^Defined as AD8 total score ≥2.

**Table 4:** AD8 subitems in dementia and nondementia subjects.

AD8 subitems	Dementia (AD8 ≥ 2) *n* = 260	Nondementia (AD8 < 2) *n* = 1284	*P* value^*^	Total *n* = 1544
Mean age	82.8 ± 8.5	73.9 ± 10.4	<0.001	75.4 ± 10.6
AD8-1	82, 31.5%	5, 0.4%	<0.001	87, 5.6%
AD8-2	110, 42.3%	32, 2.5%	<0.001	142, 9.2%
AD8-3	86, 33.1%	16, 1.2%	<0.001	102, 6.6%
AD8-4	108, 41.5%	34, 2.6%	<0.001	142, 9.2%
AD8-5	70, 26.9%	4, 0.3%	<0.001	74, 4.8%
AD8-6	67, 25.8%	2, 0.2%	<0.001	69, 4.5%
AD8-7	123, 47.3%	15, 1.2%	<0.001	138, 8.9%
AD8-8	216, 83.1%	156, 12.1%	<0.001	372, 24.1%
Mean AD8 score	3.3 ± 1.7	0.2 ± 0.4	<0.001	0.73 ± 1.39

AD8-1: problems with judgment; AD8-2: reduced interest in hobbies/activities; AD8-3: repeats questions, stories, or statements; AD8-4: trouble learning how to use a tool, appliance, or gadget; AD8-5: forgetting correct month or year; AD8-6: difficulty handling complicated financial affairs; AD8-7: difficulty remembering appointments; AD8-8: consistent problems with thinking and/or memory.

^*^The *P* value was calculated by comparing the prevalence of endorsement of each item in AD8 between suspected demented and nondemented participants.

**Table 5:** The prevalence of dementia in every age group.

Age groups	Dementia (AD8 ≥ 2)	Nondementia (AD8 < 2)
<50	0, 0.0%	30, 100%
50–59	2, 3.0%	64, 97.0%
60–64	9, 4.5%	189, 95.5%
65–69	12, 6.5%	173, 93.5%
70–74	20, 7.6%	244, 92.4%
75–79	24, 13.4%	155, 86.6%
80–84	50, 23.0%	167, 77.0%
85–89	96, 33.6%	190, 66.4%
≥90	47, 39.5%	72, 60.5%
Mean age	82.8 ± 8.5	73.9 ± 10.4
